# Prevalence of QTc interval prolongation and its associated risk factors among psychiatric patients: a prospective observational study

**DOI:** 10.1186/s12888-020-02687-w

**Published:** 2020-06-03

**Authors:** Zahid Ali, Mohammad Ismail, Zahid Nazar, Fahadullah Khan, Qasim Khan, Sidra Noor

**Affiliations:** 1 Department of Pharmacy, University of Peshawar, Peshawar, Khyber Pakhtunkhwa Pakistan; 2 Department of Psychiatry, Lady Reading Hospital, Peshawar, Khyber Pakhtunkhwa Pakistan; 3 Department of Pharmacy, COMSATS University Islamabad, Abbottabad Campus, Abbottabad, Khyber Pakhtunkhwa Pakistan

**Keywords:** QT interval prolongation, Prevalence, Risk factors, Torsade de pointes, Psychotropics, Psychiatry

## Abstract

**Background:**

QT interval prolongation is a growing concern worldwide, posing psychiatric patients to life-threatening fatal arrhythmias i.e., torsade de pointes. This study aimed to identify the prevalence of QT interval prolongation, its associated risk factors and prescribing patterns of QT prolonging drugs among psychiatric patients.

**Method:**

A prospective observational study was conducted that included psychiatric patients from a tertiary care hospital and a psychiatry clinic in Peshawar, Khyber Pakhtunkhwa, Pakistan. Electrocardiogram was recorded of those patients who were using psychotropic medications for ≥7 days, aged 18 years or more, and of either gender, male or female. The Fredericia correction formula was used for measuring QTc values (corrected QT). Chi-square test was applied to estimate differences between patients with or without prolonged QTc interval whereas, logistic regression analysis was performed to identify various predictors of QT interval prolongation.

**Results:**

Out of 405 patients, the QTc interval was prolonged in 23 (5.7%) patients including 1 (0.2%) patient with highly abnormal prolonged QTc interval (> 500 ms). QT drugs (91.6%), female sex (38.7%) and hypertension (10.6%) were the most common QT prolonging risk factors. Prolonged QTc interval was significantly higher among male patients (*p* = 0.007).

**Conclusion:**

In the present study, QT interval prolongation was observed in a considerable number of psychiatric patients. While, the high prevalence of QT prolonging risk factors among these patients warrants the increased risk of fatal arrhythmias. Therefore, risk assessment and electrocardiographic monitoring, and prescription of safer alternatives are highly recommended.

## Background

QT interval prolongation (QTIP) is a well-known surrogate marker for torsades de pointes (TdP), a life-threatening ventricular arrhythmia, that may result in sudden cardiac death [1–4]. QTIP is a consequence of abnormality in the ion channels of the heart such as potassium, sodium, and calcium channels [5]. Cardiac channel abnormalities may be congenital or acquired, the latter is more common and is often associated with drugs [6].

Psychiatric patients are at higher risk of drug induced TdP because majority of the psychotropic agents (antipsychotics and antidepressants) are notorious for prolonging the QT interval [7]. The risk is further enhanced in patients with other QT prolonging risk factors such as bradycardia, diabetes mellitus, hypertension, advance age, female sex, underlying heart diseases and illicit drugs use [8–10].

Psychiatric patients are often exposed to psychotropic polypharmacy [11, 12], high dose therapy and illicit drug use, which considerably increase the probability of exposure to QT prolonging drugs and QT drug-drug interactions (QT-DDIs) [10, 13]. In psychiatry settings, polypharmacy is a traditional and old practice that is increasingly becoming a norm rather than exception. In a review on polypharmacy in psychiatry, Kukreja et al. reported the prevalence of psychiatric polypharmacy (≥2 drugs) between 13 and 90% [14]. Another study reported, 19% prevalence of multi-class psychotropic polypharmacy among children and adolescents [15]. According to a study, since 1974 prescriptions containing ≥3 drugs increased from 5% to 40% in 1995 [16].

In addition, among psychiatric patients, the higher risk of QTIP is also due to frequent QT prolonging antidepressant and antipsychotic combinations [4, 17]. In most clinical conditions, > 1 psychotropic drug is indicated [18, 19]. In some clinical situations, compared to a single antidepressant or antipsychotic drug, patients with psychiatric illnesses significantly improved after adding a second psychotropic drug in the prescription [20–23]. However, another study also reported significant increase in psychotropic polypharmacy in a psychiatry setting with many combinations of unproven efficacy that are not supported by controlled clinical trials [11]. Recently, a study reported higher prevalence of contraindicated combinations with two antidepressants (escitalopram or citalopram) among hospitalized patients [24]. Using > 1 QT prolonging medications simultaneously increase the risk of life-threatening ventricular arrhythmias [25].

Psychiatric polypharmacy, psychotropic QT prolonging drug combinations and high dose therapies are inevitable in such a population due to multiple illnesses and tolerance to the recommended dose of therapy [13, 26, 27]. The increased prevalence of polypharmacy, contraindicated combinations and high dose therapy coupled with poor access to health care facilities increase the risk of QTIP associated morbidity and mortality [14, 24, 28].

Despite considerable safety concern for QTIP associated with psychotropic drugs, studies are scarce regarding the prevalence of QTIP and its associated risk factors among psychiatric patients, particularly in the developing countries. Moreover, electrocardiographic monitoring for QTIP is not performed in routine clinical practice despite the known risk of QTIP and TdP with psychotropic agents [9, 29]. Therefore, this study aimed to identify the prevalence of QTIP, its associated risk factors and prescribing patterns of QT prolonging drugs in psychiatric patients.

## Methods

### Design and settings

A prospective observational study was conducted at the psychiatry ward of a tertiary care hospital and a psychiatry clinic of a provincial capital. The convenience sampling technique was used to include patients diagnosed with psychiatric diseases, who used psychotropic medications for ≥7 days, aged 18 years or more (adult), and of either gender, male or female from January 31, 2018 till July 30, 2018. The Institutional Review Board (IRB) of the hospital granted the ethical approval for this study. Prior to participation, a written consent was obtained from all patients.

### Data collection and analysis

Patient’s relevant data required for this study was obtained from the medical profile of individual patient. After recording ECG the following data were collected from the patient’s medical record; gender, age, main diagnosis, comorbidities other than psychiatric disorders (if any), and prescribed medications.

The corrected QT interval (QTc) was manually calculated from the patient’s ECG using the Fredericia (QTcF) [30], and Bazett’s (QTcB) correction formula (QTcB results only presented in supplementary Table S1) [31]. QT interval was measured from the start of QRS complex till the end of T wave from lead- II on the surface of ECG. The QTIP was defined as, QTc values above 450 ms and 470 ms for male and female patients, respectively [32]. Whereas, values above 500 ms were considered highly abnormal irrespective of gender [33]. CredibleMeds database was used for the identification of QT prolonging drugs and their TdP risk categories [7]. Whereas, Micromedex DrugReax® database was used for the identification of QT-DDIs [34].

### Statistical analysis

For categorical variables, chi-square test was applied to identify the differences for various variables between patients with or without QTIP. Logistic regression analysis was performed to calculate the odds ratios for various predictors of QTIP. A *p*-value of 0.05 or less was considered statistically significant. SPSS version-23 was used for statistical analysis.

## Results

This study included 405 psychiatric patients, of which 61.2% (*n* = 248) were males and 38.8% (157) were females. The mean age of patients was 34.6 ± 13.8 years while ranging from 18 to 80 years. Majority of patients were prescribed 2–3 drugs (63%), followed by 25.2% patients who were using > 3 drugs concomitantly. Of the total patients, major depression (52.1%) was the most frequent diagnosis followed by manic depressive psychosis (8.9%), panic disorder (8.4%) and psychosis (7.7%). Whereas, hypertension (10.6%) and diabetes mellitus (4.2%) were the most common co-morbidities (Table 1).

**Table 1:** General characteristics of patients

Variables	***n*** (%)^a^
**Gender**	
Male	248 (61.2)
Female	157 (38.8)
**Age (years)**	
≤ 20	81 (20)
21–30	109 (26.9)
31–40	98 (24.2)
> 40	117 (28.9)
**All prescribed drugs**	
1	48 (11.9)
2–3	255 (63)
> 3	102 (25.2)
**Diagnosis**	
Major depression	211 (52.1)
Manic depressive psychosis	36 (8.9)
Panic disorder	34 (8.4)
Psychosis	31 (7.7)
Obsessive compulsive disorder	18 (4.4)
Schizophrenia	17 (4.2)
Hypomania	13 (3.2)
Bipolar affective disorder	11 (2.7)
Substance abuse	17 (4.2)
**Co-morbid illnesses**	
Hypertension	43 (10.6)
Diabetes mellitus	17 (4.2)
Epilepsy	13 (3.2)

^a^ Percentage calculated in total of 405 patients

Of total 405 patients, 23 (5.7%) patients were found to have prolonged QTc interval, while only one patient (0.2%) had highly abnormal QTc interval (> 500 ms), and 12.3% of patients had QTc value in the borderline range. Moreover, with QTcB, we noted higher prevalence of QTIP (27.2%) than QTcF (5.7%), details of which are provided in supplementary Table S1. QTIP was significantly higher among male patients (*p* = 0.03). Whereas, no statistically significant differences were observed between the two groups i.e., patients with QTIP vs. patients with normal QT interval with respect to other clinical characteristics (Table 2). All patients (100%) with prolonged QT interval were exposed to one or more QT prolonging drugs. At least one QT-DDI was present in 9 (39.9%) patients with QTIP.

**Table 2:** Comparative analysis of patients with normal and prolonged QTc interval

Variables	QTc interval		***p-value***
	Normal (***N*** = 382) ^a^ ***n*** (%)	Prolonged (***N*** = 23) ^b^ ***n*** (%)	
**Gender**			
Male	229 (92.3)	19 (7.7)	0.03
Female	153 (97.5)	4 (2.5)	
**Age (years)**			
≤ 20	75 (19.6)	6 (26.1)	0.45
21–30	105 (27.5)	4 (17.4)	
31–40	94 (24.6)	4 (17.4)	
> 40	108 (28.3)	9 (39.1)	
**All prescribed drugs**			
1	47 (12.3)	1 (4.3)	0.48
2–3	240 (62.8)	15 (65.2)	
> 3	95 (24.9)	7 (30.4)	
**QT prolonging drugs**			
1	216 (56.5)	10 (43.5)	0.22
≥ 2	166 (43.5)	13 (56.5)	
**QT drug-drug interactions**	109 (28.5)	9 (39.9)	0.27
**Diagnosis**			
Psychosis	29 (7.6)	2 (8.7)	0.84
Manic depressive psychosis	33 (8.6)	10 (13)	0.47
Obsessive compulsive disorder	16 (4.2)	2 (8.7)	0.30
Schizophrenia	16 (4.2)	1 (4.3)	0.97
Major depression	200 (52.4)	11 (47.8)	0.67
Hypomania	13 (3.4)	0 (0)	0.36
Panic disorder	34 (8.9)	0 (0)	0.13
Bipolar affective disorder	11 (2.9)	0 (0)	0.40
Substance abuse	17 (4.9)	0 (0)	0.30
**Co-morbid illnesses**			
Hypertension	40 (10.5)	3 (13)	0.67
Diabetes mellitus	17 (4.5)	0 (0)	0.80
Epilepsy	13 (3.4)	0 (0)	0.27
**QT prolonging drug classes (ATC Code)**			
Antipsychotic (N05A)	140 (36.6)	11 (47.8)	0.28
Proton pump inhibitors (A02BC)	48 (12.6)	1 (4.3)	0.24
Antidepressant (N06A)	253 (66.2)	14 (60.9)	0.59
Other drugs	19 (5)	2 (8.7)	0.43

^a^ Percentage calculated in total of 382 patients with normal QTc interval except gender; ^b^ Percentage calculated in total of 23 patients with prolonged QTc interval except gender

Table 3 shows frequencies of QT prolonging drugs and their therapeutic classes along with their TdP risk among patients with QTIP. All patients with prolonged QTc interval (*n* = 23) were receiving QT prolonging drugs. Of which, the most frequently prescribed drug classes were antidepressants (*n* = 13), and antipsychotics (*n* = 5). Among antidepressants, 30.4% of the drugs were carrying conditional risk of TdP, and 17.4% were having known risk of TdP. Among antipsychotics, 8.6% of the drugs were having known and possible risk of TdP, respectively. Moreover, majority of the patients (65.2%) with QTIP had no QT prolonging risk factors except exposure to QT prolonging drugs and female gender, indicating the high probability of drug induced QTc interval prolongation, details have been presented in supplementary Table S2.

**Table 3:** Frequency of QT prolonging drugs and their therapeutic classes along with TdP risk categories among patients with prolonged QTc interval

Therapeutic class	TdP risk ^a^	QT prolonging drugs (ATC Code)	***n*** (%) ^b^
Antidepressants (*n* = 13)	Known (*n* = 4)	Escitalopram (N06AB10)	4 (17.4)
	Possible (2)	Clomipramine (N06AA04)	1 (4.3)
		Venlafaxine (N06AX16)	1 (4.3)
	Conditional (7)	Sertraline (N06AB06)	3 (13)
		Fluoxetine (N06CA03)	2 (8.7)
		Fluvoxamine (N06AB08)	1 (4.3)
		Paroxetine (N06AB05)	1 (4.3)
Antipsychotics (5)	Known (2)	Haloperidol (N05AD01)	1 (4.3)
		Levosulpiride (N05AL07)	1 (4.3)
	Possible (2)	Risperidone (N05AX08)	1 (4.3)
		Aripiprazole (N05AX12)	1 (4.3)
	Conditional (1)	Olanzapine (N05AH03)	1 (4.3)
Proton pump inhibitors (2)	Conditional (2)	Esomeprazole (A02BC05)	2 (8.7)
Diuretics (2)	Conditional (2)	Hydrochlorothiazide (C03AA03)	1 (4.3)
		Furosemide (C03CA01)	1 (4.3)
Antinausea (1)	Known (1)	Domperidone (A03FA03)	1 (0.9)

^*a*^ TdP risk was based on CredibleMeds® database; ^*b*^ Percentage calculated in total of 23 patients

Univariate, and multivariate logistic regression analysis demonstrated significant association of QTIP with male gender (*p =* 0.03 and *p =* 0.007, respectively) as compared with females as shown in Table 4. No statistical association was observed with other clinical characteristics (Table 4).

**Table 4:** Logistic regression analysis

Variables	Univariate analysis		Multivariate analysis	
	OR (95% CI)	***p-value***	OR (95% CI)	***p-value***
**Gender**				
Female	Reference		Reference	
Male	3.2 (1.1–9.5)	0.03	5.6 (1.6–19.8)	0.007
**Age (years)**				
≤ 20	2.1 (0.5–7.7)	0.26	2.8 (0.6–11.3)	0.15
21–30	Reference		Reference	
31–40	1.1 (0.2–4.5)	0.87	1.3 (0.2–5.9)	0.71
> 40	2.1 (0.6–7.3)	0.20	2.5 (0.6–9.1)	0.16
**All prescribed drugs**				
1	Reference		Reference	
2–3	2.9 (0.3–22.7)	0.32	5.5 (0.5–57.2)	0.14
> 3	3.5 (0.4–28.9)	0.25	8.5 (0.6–111.3)	0.10
**QT prolonging Drugs**				
1	Reference		Reference	
≥ 2	1.7 (0.7–3.9)	0.22	1.9 (0.6–5.1)	0.23
**Medical illnesses**				
Psychosis	1.2 (0.2–5.1)	0.84	5.7 (0.7–41.8)	0.08
Obsessive compulsive disorder	2.1 (0.4–10.1)	0.32	2.3 (0.2–19.9)	0.45
Schizophrenia	1.04 (0.1–8.2)	0.97	8.2 (0.7–95.1)	0.09
Major depression	0.8 (0.3–1.9)	0.67	3.8 (0.7–20.4)	0.11
Manic depressive psychosis	1.6 (0.4–5.6)	0.47	2.3 (0.3–13.6)	0.35
**Co-morbid illnesses**				
Hypertension	1.3 (0.3–4.5)	0.69	0.5 (0.1–2.9)	0.50
Antipsychotics	1.6 (0.6–3.6)	0.28	1.9 (0.4–9.3)	0.38
Proton pump inhibitors	0.3 (0.04–2.4)	0.26	6.6 (0.7–62.5)	0.09
Other drugs	1.8 (0.3–8.3)	0.44	0.6 (0.1–3.9)	0.59

## Discussion

This present study found QTIP in 5.7% of the patients that is higher in comparison to studies conducted in developed countries [35–40]. The study also identified highly abnormal QTIP (> 500 ms) in 0.2% of patients which is lower than studies conducted in Japan (1.2 and 3%) [41, 42], Italy (2.3%) [9], Spain (2%) [43], and Switzerland (0.9%) [39]. These inconsistencies in prevalence rates may be attributed to variability in the study population and drug prescribing/utilization patterns among respective countries. On the contrary, we observed higher prevalence of QTIP with QTcB, details of which are presented in supplementary Table S1 because majority of studies conducted in psychiatry settings have used QTcB [9, 39, 41–43]. Therefore, health care professionals should be more vigilant regarding the assessment and prevention of QTIP among psychiatric patients.

The risk of QTIP was 5.6 times higher in males than in female patients, which is in contrast with the findings of other studies [44, 45]. However, some studies have reported mixed results regarding the prevalence of QTIP with respect to gender [46, 47]. This high prevalence of QTIP in males may be attributed to high frequency of prescribed QT prolonging drugs. In the present study, no statistically significant association of QTIP were observed with age, multiple drugs, ≥2 QT prolonging drugs, medical and co-morbid illnesses, which are not in agreement with other studies [8, 27]. This variation in results may be due to small sample size, and lower mean age of study participants (34.6 years).

A high proportion of patients were exposed to QT prolonging risk factors, of which QT prolonging drugs were most common, followed by female gender and hypertension. Almost 92% of patients were exposed to QT prolonging drugs, of which, considerable number of patients were taking multiple QT prolonging drugs concomitantly. While, literature suggests avoiding concomitant use of antidepressants and antipsychotics, when possible, but it is usually impossible in psychiatric population [27]. Therefore, proper risk assessment and vigilant monitoring is recommended to identify patients at low/high risk of QTIP. Risk assessment tools as developed by Vandael et al. [48] should be integrated in routine practice in psychiatry wards in order to develop a risk score on the basis of which patients will be either included or excluded for further follow up.

Although, the present study highlighted an important area of patients’ safety, but there are some limitations which need to be addressed in future studies. Following are the potential limitations of this study such as this study was conducted in a single city which may limit the generalizability of the findings. Therefore, a multicenter study in future may provide a better insight regarding this issue. We did not assess the association of QTIP with duration of illness, and the duration of use of antipsychotics and antidepressants. Moreover, in our study the sample size for major depression was high compared to other psychiatric disorders which may lead to potential bias, therefore, inclusion of patients with equal frequency of other psychiatric disorders may address this issue. Further, we used CredibleMeds® list of QT prolonging drugs as a reference in order to identify prescribed QT prolonging drugs. This database has its own criteria for the inclusion of QT drugs, hence some drugs other than the enlisted ones might be having QT prolonging effect.

## Conclusions

The present study identified QTIP in considerable number of patients. A high proportion of psychiatric patients were exposed to QT prolonging risk factors, of which, QT prolonging drugs were most common. Therefore, proper consideration should be offered to the assessment of QTIP and selection of drug therapy in these patients (male patients in particular) in the presence of QT prolonging risk factors in order to ensure patients’ safety and prevent life-threatening cardiac arrhythmias.

## Supplementary information

**Additional file 1: Table S1.** Comparative analysis of patients with normal and prolonged QTc interval using Bazett’s correction formula.

**Additional file 2: Table S2.** Cases with high probability of drug induced QT interval prolongation.

## Data Availability

The datasets used and/or analyzed during the current study are available from the corresponding author on reasonable request.

## Abbreviations

IRB: Institutional Review Board;
QTc: Corrected QT interval
QT-DDIs: QT drug-drug interactions
QTIP: QT interval prolongation
TdP: Torsade de pointes
