# Supplementary material for: Prevalence of QTc interval prolongation and its associated risk factors among psychiatric patients: a prospective observational study
Source: BMC Psychiatry. 2020 Jun 3;20:277. doi: 10.1186/s12888-020-02687-w (PMC7268705; doi:10.1186/s12888-020-02687-w)
Supplement: Supplementary file 1 — Additional file 1: Table S1. Comparative analysis of patients with normal and prolonged QTc interval using Bazett’s correction formula. [file 12888_2020_2687_MOESM1_ESM.docx]

**Supplementary Table S 1: Comparative analysis of patients with normal and prolonged QTc interval using Bazett’s correction formula**

| **Variable** | **QT interval** | | **Chi-square Test** | **Univariate analysis** | |
| --- | --- | --- | --- | --- | --- |
|  | **Normal (N=295)** ^a^  ***n* (%)** | **Prolonged (N=110)** ^b^  ***n* (%)** | ***p-value*** | **OR (95% CI)** | ***p-value*** |
| **Gender** |  |  |  |  |  |
| Male | 182 (73.4) | 66 (26.6) | 0.75 | Reference |  |
| Female | 113 (72) | 44 (28) |  | 1.1 (0.7-1.7) | 0.75 |
| **Age (years)** |  |  |  |  |  |
| ≤20 | 59 (20) | 22 (20) | **0.005** | 2 (1-4.1) | **0.05** |
| 21-30 | 92 (31) | 17 (15.5) |  | Reference |  |
| 31-40 | 70 (23.7) | 28 (25.5) |  | 2.2 (1.1-4.3) | **0.02** |
| >40 | 74 (25.1) | 43 (39.1) |  | 3.1 (1.6-6) | **< 0.001** |
| **All prescribed drugs** |  |  |  |  |  |
| 1 | 43 (14.6) | 5 (4.5) | **0.02** | Reference |  |
| 2-3 | 181 (61.4) | 74 (67.3) |  | 3.5 (1.3-9.2) | **0.01** |
| >3 | 71 (24.1) | 31 (28.2) |  | 3.8 (1.4-10.4) | **0.01** |
| **QT prolonging drugs** |  |  |  |  |  |
| 1 | 150 (50.8) | 42 (38.2) | 0.07 | Reference |  |
| ≥2 | 121 (41) | 58 (52.7) |  | 1.7 (1.1-2.7) | **0.02** |
| **QT drug-drug interactions** | 84 (28.5) | 34 (30.9) | 0.63 | - | - |
| **Diagnosis** |  |  |  |  |  |
| Psychosis | 18 (6.1) | 13 (11.8) | **0.05** | 2.1 (0.9-4.3) | **0.05** |
| Manic depressive psychosis | 26 (8.8) | 10 (9.1) | 0.93 | 1.8 (0.7-4.6) | 0.25 |
| Obsessive compulsive disorder | 11 (3.7) | 7 (6.4) | 0.25 | 1.1 (0.4-3.3) | 0.83 |
| Schizophrenia | 12 (4.1) | 5 (4.5) | 0.83 | 1 (0.6-1.5) | 0.94 |
| Major Depression | 154 (52) | 57 (51) | 0.94 | 1 (0.5-2.2) | 0.93 |
| Hypomania | 11 (3.7) | 2 (1.8) | 0.33 | 0.8 (0.3-1.8) | 0.61 |
| Panic disorder | 26 (8.8) | 8 (7.3) | 0.61 | 0.5 (0.1-2.1) | 0.34 |
| Bipolar affective disorder | 10 (3.4) | 1 (0.9) | 0.17 | 0.3 (0.03-2.1) | 0.20 |
| Substance abuse | 15 (5.1) | 2 (1.8) | 0.14 | 0.3 (0.08-1.5) | 0.16 |
| **Co-morbid illnesses** |  |  |  |  |  |
| Hypertension | 26 (8.8) | 17 (15.5) | **0.05** | 1.9 (0.9-3.6) | **0.05** |
| Diabetes mellitus | 13 (4.4) | 4 (3.6) | 0.73 | 0.8 (0.2-2.5) | 0.73 |
| Epilepsy | 10 (3.4) | 3 (2.7) | 0.73 | 0.7 (0.2-3) | 0.73 |
| **QT prolonging drug classes (ATC Code)** |  |  |  |  |  |
| Antipsychotic (N05A) | 104 (35.3) | 47 (42.7) | 0.16 | 0.8 (0.5-1.3) | 0.40 |
| Proton pump inhibitors (A02BC) | 33 (11.2) | 16 (14.5) | 0.35 | 1.3 (0.9-2.1) | 0.16 |
| Antidepressant (N06A) | 198 (67.1) | 69 (62.7) | 0.40 | 1.3 (0.7-2.6) | 0.35 |
| Other drugs | 14 (4.7) | 7 (6.4) | 0.51 | 1.4 (0.5-3.5) | 0.51 |

^a^ Percentage calculated in total of 295 patients with normal QTc interval except gender; ^b^ Percentage calculated in total of 110 patients with prolonged QTc interval except gender
