# Supplementary material for: Prevalence of QTc interval prolongation and its associated risk factors among psychiatric patients: a prospective observational study
Source: BMC Psychiatry. 2020 Jun 3;20:277. doi: 10.1186/s12888-020-02687-w (PMC7268705; doi:10.1186/s12888-020-02687-w)
Supplement: Supplementary file 2 — Additional file 2: Table S2. Cases with high probability of drug induced QT interval prolongation. [file 12888_2020_2687_MOESM2_ESM.docx]

**Table S 1: Cases with high probability of drug induced QT interval prolongation**

| **Gender** | **n (%)**^a^ |
| --- | --- |
| Male | 15 (100) |
| Female | 0 (0) |
| **Diagnosis** |  |
| Major Depression | 11 (73.3) |
| Psychosis | 2 (13.3) |
| Manic depressive psychosis | 2 (13.3) |
| **QT prolonging drugs** |  |
| Antidepressants | 9 (60) |
| Antipsychotics | 7 (46.7) |
| Proton pump inhibitors | 1 (6.7) |
| **TdP risk of drugs** |  |
| Known risk of TdP | 7 (46.7) |
| Possible risk of TdP | 3 (20) |
| Conditional risk of TdP | 5 (33.3) |

^a^Percentage calculated in total of 15 patients having no QT prolonging risk factors and prescribed with QT prolonging drugs
